# Cash transfer interventions for sexual health: meanings and experiences of adolescent males and females in inner-city Johannesburg

**DOI:** 10.1186/s12889-018-5027-3

**Published:** 2018-01-10

**Authors:** Nomhle Khoza, Jonathan Stadler, Catherine MacPhail, Admire Chikandiwa, Heena Brahmbhatt, Sinead Delany-Moretlwe

**Affiliations:** 1 Wits Reproductive Health and HIV Institute (Wits RHI), Faculty of Health Sciences, School of Clinical Medicine, University of the Witwatersrand, Johannesburg, South Africa; 2 School of Health and Society, University of Wollongong, Wollongong, NSW Australia; 3 Department of Population Reproductive and Family Health, John Hopkins Bloomberg School of Public Health, Baltimore, USA

**Keywords:** Cash transfers, Adolescents, Girls, Boys, South Africa

## Abstract

**Background:**

In sub-Saharan Africa, there is growing interest in the use of cash transfer (CT) programs for HIV treatment and prevention. However, there is limited evidence of the consequences related to CT provision to adolescents in low-resourced urban settings. We explored the experiences of adolescents receiving CTs to assess the acceptability and unintended consequences of CT strategies in urban Johannesburg, South Africa.

**Methods:**

We collected qualitative data during a pilot randomized controlled trial of three CT strategies (monthly payments unconditional vs. conditional on school attendance vs. a once-off payment conditional on a clinic visit) involving 120 adolescents aged 16–18 years old in the inner city of Johannesburg. Interviews were conducted in isiZulu, Sesotho or English with a sub-sample of 49 participants who adhered to study conditions, 6 months after receiving CT (280 ZAR/ 20 USD) and up to 12 months after the program had ended. Interviews were transcribed and translated by three fieldworkers. Codes were generated using an inductive approach; transcripts were initially coded based on emerging issues and subsequently coded deductively using Atlas.ti 7.4.

**Results:**

CTs promoted a sense of independence and an adult social identity amongst recipients. CTs were used to purchase personal and household items; however, there were gender differences in spending and saving behaviours. Male participants’ spending reflected their preoccupation with maintaining a public social status through which they asserted an image of the responsible adult. In contrast, female participants’ expenditure reflected assumption of domestic responsibilities and independence from older men, with the latter highlighting CTs’ potential to reduce transactional sexual partnerships. Cash benefits were short-lived, as adolescents reverted to previous behavior after the program’s cessation.

**Conclusion:**

CT programs offer adolescent males and females in low-income urban settings a sense of agency, which is vital for their transition to adulthood. However, gender differences in the expenditure of CTs and the effects of ending CT programs must be noted, as these may present potential unintended risks.

## Background

Cash transfer (CT) programs are largely used in low- and middle-income countries as a mechanism to alleviate poverty. In the past two decades, they have been used to improve health outcomes and promote healthy behavior. Studies show that conditional and unconditional CTs have significantly improved health-seeking behaviors and the uptake of healthcare services [1, 2]. Following the impact of CT programs on improved health outcomes in Latin America, there has been a growing interest in their use for HIV treatment and prevention [3]. It is hypothesized that through mitigation of the underlying economic drivers of HIV infection or incentivization of healthy behavior, CT programs could lessen engagement in risky behavior, thereby reducing HIV infections [4, 5].

Studies in developing countries show promising findings in relation to the potential role of CTs in reducing HIV infection by changing risky sexual behaviors [3]. Studies have also shown that CT programs could support HIV treatment by enabling recipients living with HIV to buy food and pay for costs associated with treatment access, thereby improving adherence to antiretroviral therapy (ART) and retention in HIV care [6, 7]. There are also debates about the potential role of CTs to improve utilization of and retention in mother-to-child transmission services (PMTCT) [8]. CTs conditional on prenatal clinic visits increased retention in the PMTCT cascade and uptake of prenatal services [9]. While CT programs are a promising mechanism to leverage positive HIV programme outcomes, the effectiveness of interventions may vary according to the demographic attributes of the target population [10, 11].

CT programs have historically targeted adult women [12] and there is limited evidence on the impact of CT programs where cash is given directly to adolescents. Despite this limited evidence, there is a growing interest in expanding social protection programs in the African region and in using CTs to improve HIV outcomes in adolescent girls and young women (AGYW), given this group’s high incidence of HIV infection [13]. Research findings to support the effectiveness of this approach are limited, but promising [14]. An observational study in South Africa showed that adolescent girls from households that were receiving a child support grant were less likely to engage in intergenerational sex, which is a risk factor for HIV infection [15]. A randomized controlled trial (RCT) in Malawi showed that AGYW who received school-conditioned CTs were less likely to become pregnant, marry early, and engage in risky sexual behavior [16]. A recent South African study however showed that, while CTs conditional on school attendance reduced risk behaviors in adolescent girls, this did not translate into reductions in HIV incidence [17]. Similarly, another trial also in South Africa found that cash incentives conditional to 80% participation in a life skills program, taking an HIV test, passing academic tests, and submitting community project report, had no effect on HIV incidence, but reduced HSV-2 incidence [18].

Despite the inconsistent findings, agencies such as UNAIDS and the World Bank are willing to support governments to scale up CT programs [19]. The United Nations Development Program (UNDP) has recommended further exploration of CTs with regard to their potential to prevent HIV among key populations [11]. Moreover, Swaziland’s Ministry of Health is considering scaling up the implementation of CCT programs to prevent HIV infection among AGYW [20].

While AGYW are a priority target group for these sorts of structural interventions to address HIV; Gibbs et al. (2012) highlight the need to include men in structural interventions for HIV prevention [21], as evidence shows an association between men’s income, their views of manhood and the risk of HIV [22, 23]. The pathways through which perceptions of manhood and livelihood influence HIV remain unclear [21]. Moreover, studies indicate that CT interventions may impact men and women in different ways [24]. While CTs may encourage healthy behaviors and elicit intended behaviors among recipients [3], CTs may also encourage unanticipated negative behaviors such as alcohol and drug use, which in turn increase the recipients’ health risks.

Currently, it is not known how adolescents in a low-resourced African urban setting perceive CTs, how they spend CTs, or what receipt of transfers means to them. In addition, there is limited data and understanding regarding the impact of cash transfers on adolescent male behaviours. This limits the understanding of the impact of CT programs designed for HIV prevention and treatment in various contexts, indicating the need for further research in this regard. We present findings from a qualitative study conducted among adolescent boys and girls who participated in a CT intervention study where we explored participants’ experiences of the CT intervention and the meaning that they attach to the intervention.

## Methods

### Study population and setting

The research was conducted in inner city of Johannesburg, South Africa, specifically in the Hillbrow, Berea, and Yeoville areas. This location was selected because of evidence that HIV prevalence in South Africa is highest in informal urban areas [13, 25]. The area is characterized by dense, overcrowded high-rise residential buildings and abandoned industrial buildings informally occupied by migrant workers from within and outside South Africa. Fifty-one percent of the population in the area are males, 20% are aged <15 years, and 21% are aged 15–24 years. The area is characterized by a high concentration of low-income households (<R1000 per month) and unemployment rate of 41% (Statistics South Africa, 2012 as cited in USAID report [26]).

### Study design

This study was nested within the *CHANGE* study*,* a pilot RCT assessing the feasibility and acceptability of CTs in a low-resourced urban setting. The aim of the intervention was to compare three different CT interventions strategies.

We recruited male and female adolescents (*n* = 120) for participation in the intervention through distribution of pamphlets at three schools and by word of mouth. The inclusion criteria were being aged 16–18 years, self-identifying as sexually active, and enrolled in school. Potential participants were contacted and invited to our research offices (along with a parent/ legal guardian, for those aged 16–17 years, who in the context of participation in public health research are still considered minors).

Participants were randomized into one of three cash strategies using sealed numbered envelopes: 1) Unconditional monthly payments of 280 ZAR (USD 20) for 6 months; 2) monthly payments of 280 ZAR for 6 months, conditional on 80% school attendance; 3) and a single payment of 280 ZAR conditional on a once-off clinic visit involving sexual reproductive health education, services related to family planning and contraception, HIV counselling and testing, HIV risk assessment, and HIV risk reduction counselling. Payments were made through a cardless service that allowed cash withdrawals from an automated teller machine (ATM) using a pin code, which was sent to participants’ mobile phones via a short message system (SMS). Those who either did not have a mobile phone or had a shared phone were paid using ATM cards with a linked pin; these were provided to participants at enrollment. Participants completed surveys at 6 and 12 months after receiving CTs; the survey results are reported elsewhere [26].

### Sample and data collection procedures

From the 120 participants enrolled in the intervention, we purposively selected a sub-sample of 49 adolescent boys (*n* = 30) and girls (*n* = 19) to participate in-depth interviews. We selected participants who adhered to study conditions such as attending school and visiting the clinic across the three study arms (i.e., unconditional cash transfers, clinic-conditioned cash transfers, and school-conditioned cash transfers).

Data were collected 6 months after participants had received the conditional and unconditional CTs (March 2014) and up to 12 months after the intervention had ended (March 2015). Different sets of participants were interviewed at the two time points. The purpose of the second interviews was to gain insights into the effects after the cessation of a CT programme. We contacted participants telephonically and invited them to take part in the interviews.

Participants were interviewed in a private room at our research offices in Hillbrow. The interviews were conducted by one male and two female interviewers, who were fluent in isiZulu and Sesotho. Each interview took approximately 1 h, and participants were compensated 100 ZAR (approximately USD 7) for transport costs and their time. The interview guide consisted of the following topics: (1) descriptions of adolescents’ households (household membership, employment, domestic relationships); (2) experiences of the CT program; (3) participants’ sources of income before, during, and after the CT intervention; (4) and spending patterns and perceived impact of CTs. All interviews were audio-recorded and transcribed. Interviews in isiZulu and Sesotho were transcribed and translated (one-step) into English by trained transcribers. Personal identifiers were substituted with pseudonyms during transcription.

### Data analysis

An interpretative phenomenological approach was employed to explore how adolescents make sense of their experiences of CT programs. The phenomenological approach focuses on how people perceive and talk about objects or events [27]. This method was useful in facilitating an understanding of adolescents’ experiences and meanings of CT programs.

As an initial step, two of the authors of this paper (JS and NK) reviewed the transcripts to identify preliminary themes and sub-themes, using an interpretivist approach to analyze the data [27]. Atlas.ti version 7.4 was used to code and organize the transcripts. A codebook was generated using an inductive approach; transcripts were initially coded based on emerging themes and subsequent transcripts were coded deductively. The final code frame consisted of 61 codes. These codes were grouped into five thematic categories, namely, demographic information, perceptions and experiences of CT, spending patterns, meanings of CT, and developments after the CT program. After forming the thematic categories, a comparison between males and females was done. Thereafter, common themes were identified, compared and contrasted within and between cases.

## Results

### Socio-demographic characteristics

We had intended to include 30 participants at each follow up, making up an envisaged total of 60 participants. However, obtaining a balanced representation according to gender was challenging, especially at the 12-month visit. Some of the contact information was invalid and some participants had completed school, while others had relocated. Our final sample of 49 participants, therefore, had more male participants and more participants in the unconditional arm than was initially planned. Table 1 illustrates the final study sample.

**Table 1:** Demographic characteristics of young men and women who participated in IDIs in the *CHANGE * trial

*Demographic characteristics*	Sample at 6 months *n* (%)	Sample at 12 months *n* (%)	Total sample: 6 and 12 months *n* (%)
*Age (years)*	16–18	17–19	16–19
*Gender*			
Male	9 (47)	21 (70)	31 (64)
Female	10 (53)	9 (30)	18 (37)
*Study arm*			
Unconditional cash transfers	8 (42)	13 (43)	21 (43)
Clinic-conditioned cash transfers	5 (26)	8 (27)	13 (27)
School-conditioned cash transfers	6 (32)	9 (30)	15 (31)
Total	19 (100)	30 (100)	49 (100)

The majority (*n* = 21) of participants were recipients of unconditional CTs, followed by 15 participants who formed part of the school arm, and 13 participants in the clinic arm. At 6 months follow-up, the age range was 16–18 years. Participants were in grades 10–12. At 12 months follow-up, the majority (67%) of participants had completed the 12th grade; a few (13%) participants were enrolled in tertiary education, some (30%) worked temporary jobs (including informal economy), and others (23%) were seeking employment. Most participants’ households were headed by single parents, who were in unskilled employment or ran informal businesses. The majority of participants lived in one-bedroomed apartments. On average, there were four occupants per household, with parents sleeping in the bedroom and siblings sharing a bed in the living room. In some instances, two or three families shared a living space.

### Experiences of the CT intervention

At the beginning of the CT program, both adolescent boys and girls doubted its authenticity. “Receiving money for doing nothing” seemed too good to be true; this perception was also due to the high rate of scams and crime in the study area. P9 (male, school) stated: *“At first, I didn’t trust it; I thought it was a scam because, how can someone give you R250* [R280] *... just giving you just like that?”* Some of the female participants thought that they would be expected to do something in return for CTs. However, the mistrust and uncertainties diminished upon adolescents obtaining more information about the intervention and receiving their payments.

Overall, the unconditional and conditional CTs were acceptable to both boys and girls, who indicated that the program encouraged them to be responsible and attend school, and prevented girls from engaging in sex with older men, and boys from committing crime to get money. As one participant noted: *“…at least there is a project out there that is willing to help young girls and guys and show them that they don’t have to do bad things that are out of their comfort zone to get money.”* (P19, female, unconditional).

While the study did not offer formal lessons on sexual reproductive health (SRH), several participants mentioned that they liked the information on HIV/AIDS and sexually transmitted infections, which they received through their participation in the study. In relation to this, P8 (male, unconditional) illustrated: *“For me, being in the program was all about learning about HIV and how to protect ourselves from contracting such diseases by practicing safe sex.”* Similar to other participants, P8 reported that he gained knowledge about HIV and testing by completing a mobile phone-based survey in the main trial.

#### Experiences of the CT conditionalities

We asked participants about their experiences of the conditionality of CTs. Both adolescent boys and girls were open to the school condition, since they had been attending school before the program; so, being compensated for this with cash was an added advantage. As P32 (female, school) noted: *“I was going to school every day. It is something that I was doing since grade 1; so, it was not like I was working for it, because it was something that I was already doing. So, I was just getting paid for something that I was doing, but I was now pushing even harder because I was getting paid for it.”* Several adolescents in the school arm reported that CTs had minimal to no impact on their school attendance. Some indicated that the program encouraged them not to miss school. They put in extra effort into their attendance, as they did not want to miss their cash payments; for example, they attended school even when they were sick. However, this was contradictory to the main trial’s findings, which showed that participants who received school-conditioned payments were more likely to report missing school relative to participants in the other study arms [28].

Among adolescents in the clinic condition arm, several girls reported that they disliked clinic visits because of the stigma associated with being at the clinic, but that the friendly clinic staff had put them at ease. Receiving clinic-conditioned CTs encouraged participants to attend the clinic, as per study requirements, which offered the adolescents, particularly boys, an opportunity to get tested for HIV, something that they would be less likely to do if they were not receiving conditional CTs.

*“Since I got the clinic part of the study, what I saw is that they gave me a chance to go to the clinic and actually get myself tested or just do anything that I wanted to do, and that has never come to mind. I got the chance to know my status and also get counseling and be taught more about STIs.”* (P14, male, clinic).

#### The size and frequency of the CT payment

Nearly all participants, boys and girls, were satisfied with the sum of the CT (280 ZAR). They stated that the amount was “more than enough”; some described it as “*neither too small nor too much.*” It was sufficient to cover their needs. P5 (male, unconditional) remarked: *“It was a lot because I am not used to having that amount of money.”* For the adolescents, the money was also sufficient because it was not common for them to receive such money without having worked for it. Only two participants articulated that the grant was insufficient, “*this money was not enough for me … because there are things I thought I would do but the money was not enough.*” suggesting that they should have received more or the same amount on a weekly basis. Some participants stated that if we offered adolescents more money, they would misuse it. P13 (female, unconditional) commented: *“It was a good amount because knowing us, youth, we would buy alcohol. With two-eighty (280), we could buy necessities, and not things that we want, but things that we needed.”*

While adolescents were generally satisfied with the amount provided, the frequency with which the cash payments were made mattered. Participants across the three study arms stated that the program was unfair to those who had received once-off payments. Adolescents who had received once-off payments raised a concern especially in relation to peers who misused the CTs on expensive items when there were “deserving” participants who would have done anything to get the money. The assumption was that those who “need” the cash were more likely to spend it in a manner perceived to be sensible compared to those for whom the transfer was extra income.

#### The payment method

Adolescents received their payments using a bank card or a cardless system at an ATM. They liked both payment methods, reporting that receiving their payments was stress-free. Participants who utilized the card method stated that they could withdraw money from any ATM, and those who used the cardless method said that they did not have to carry cards and wallets around, which they regarded as a safe option. Making ATM transactions offered participants a sense of maturity. However, several reported that they could not withdraw the entire amount; so, they bought airtime through the ATM to use amounts that were under the limit for withdrawals.

### Patterns of spending CTs

Both adolescent boys’ and girls’ spending and saving patterns reflected the desire to look and feel good about themselves. Adolescents mostly used CTs to meet personal and household needs. Typical personal spending was on personal care/ hygiene products, clothing, school necessities, airtime, short-term savings, and social activities such as attending a school dance. Spending also included contribution towards groceries or helping others, such as giving a parent money for transport to work or buying a t-shirt for a sibling. Table 2 illustrates typical examples of spending behaviour.

**Table 2:** Typical examples of adolescents’ spending behavior

Intervention arm (amount & duration)	Participant ID, gender	Items bought/ activities paid for	Amount spent (ZAR)
School attendance condition (280 ZAR for 6 months)	P41, male	Gym	200
		Nutritional supplements Airtime	400
		Toiletries	50
		Pocket money	50
		Savings	^a^
	P20, male	Going out with friends	^a^
		Clothes	800
		Petrol	350
		Movies	200
	P1, female	Transport to school & lunch	100
		Shoes	80
		Savings (school holidays)	100
		Household groceries	^a^
	P6, female	School jersey	100
		Household groceries	100
		Transport for her mother	^a^
		Stationery	^a^
		Pocket money	^a^
		Airtime	30
Unconditional (280 ZAR for 6 months)	P26, male	Clothes for matric dance	1330
		Entry fee for matric dance	350
	P20, male	Eating out	^a^
		Movies	350
		Petrol	350
		Gym supplements	^a^
		Gym gear	^a^
		Clothes	800
		Airtime	^a^
	P13, female	Savings (matric dance)	^a^
		Birthday gift	^a^
		Household groceries	^a^
	P10, female	Other personal items	100
		Household electricity	50
		Cosmetics	50
		Snacks	50
		Airtime	30
		Picnic	50
Clinic visit condition (280 ZAR once-off payment)	P21, male	Gym	180
		Airtime	^a^
	P27, male	Entry fee & clothes for matric dance	260
		Lunch at school	20
	P4, female	Gave her mom for groceries	140
		Movies	60
		Savings (trip with friends)	50
		Cosmetics (perfume, makeup)	40
	P3, female	Shoes	^a^
		Savings (cellphone)	60
		Lunch at school	60
		Airtime for her siblings	50

^a^Indicate that exact amount was not specified

Most adolescents stated that CTs allowed them to pay for activities and items that their parents would not buy them or pay for; these included expensive clothing, make-up, mobile phones, and entertainment. In some instances, participants could purchase essential items that parents could barely afford; for example, a “school jersey, food in the house, and toiletries.” Not much spending was reported on school items, and one young woman reported spending cash on transport to the clinic and medication. Four participants, male and female, mentioned that they used CTs to buy condoms to protect themselves, mentioning that it was difficult for them to access free condoms at the clinic because of stigma.

We noted gender differences in the adolescents’ decision making regarding the use of CTs and some differences in their spending and saving patterns. Most girls sought advice from their parents or other family members on how to use the money. Some girls believed that they had to discuss this with their parents first, even though they made the ultimate decision. The most common advice that they received from parents was to spend the money wisely, and some were encouraged to save it. In some instances, mothers used and controlled the CT, but even then, this had been the participants’ decision.

“I sat down with my mom and told her that I have got the money, and she asked what I have decided to do with it, and I told her that I would give her half of the money to buy what she needs in the house, while I would use the other half to buy toiletries for myself.” (P4, female, clinic)

Contrary to girls, most boys made independent decisions regarding expenditure. They did, however, mention discussing the use of CTs with their peers. Boys were also most likely to spend CTs on airtime, mobile phones and social activities such as “going out with the boys” and the school dance, which was quite a popular and expensive event. They also spent CTs on clothing, gym, and technology. P18, a young man enrolled in the school-conditioned arm, described how he spent the money each month:

“I just spent the first payment on useless things, going to the movies with friends. Then, I used it to go to the camp. The second payment, I asked my mom to add more money onto it, and I bought sneakers for R450 … the third month … that was when I wanted a phone. I asked my mom to add more money onto it, then I purchased a phone for R500 … the fourth payment was like the first payment … I just spent it; I just bought anything I saw that I was interested in ... it was like earphones and a memory card and all that. The last two months ... that’s when I saved my money.”

In contrast to the above, girls spent some of their CTs on their personal necessities and shared some with family members. They mostly spent the cash on domestic necessities, for example, buying food, helping their parents with transport money, and helping siblings with pocket money. They also paid for personal necessities such as personal care/hygiene products, savings, and clothing. P6, a girl who had received school-conditional payments, illustrated how she spent her monthly payments:

“… the first payment, I bought a school jersey, then the second payment, there were things that we needed in the house; so, I bought the things that we needed. At other times I lent to my mom if she did not have money for transport to go to work. When others had money, they liked [to buy] Kentucky [fried chicken]; so, I would go and buy vegetables or a tray of chicken, and go home, cook, and eat with my mom.”

Generally there were similarities in patterns of expenditure across intervention arms. Similar to girls who had received monthly payments for 6 months, those who had received a single payment also spent CTs on their personal needs and to support family members.

“I gave half of the money to my mom to buy groceries in the house, and then I used some of the money for entertainment. I went to the movies with my friends, and I used some of it for toiletries like perfume, make-up and other things that I needed.” (P4, female, clinic)

Only two male participants reported spending CTs on alcohol, to entertain themselves. However, adolescents highlighted that expenditure of CTs on alcohol was common among their friends and acquaintances. They spoke of peers who used CTs on “alcohol and drugs” and “gambling,” and “falling prey to money lenders,” which reflected some form of moral judgment. *“Most of the young people that I know used their money for drugs and alcohol.”* (P20, male, unconditional).

We also noted that the meanings attached to CTs could influence how adolescents spent the money. Some participants in the unconditional arm considered CTs “free money” because they “did not work for it” and there were no conditions for receiving the cash. This is how P44, a young man, perceived unconditional CTs: *“To me, it was just free money. (Laughs) So, it didn't matter ... like, it was just free money.”* Some participants who had received a once-off payment viewed it as “luck money”; one participant in the clinic arm described it as money that you pick up on the street. The notion of “free money” or “luck money” yielded two possibilities among participants. For most, free money was supposed to be saved or spent wisely on necessary items, and for a few, it was to be spent randomly and not carefully planned for.

### The meaning of CTs for adolescents

#### Financial autonomy

One of the most prominent themes cited by the adolescent boys and girls in this study was the sense of economic independence that they experienced upon receiving CTs. P43 (male, unconditional) remarked: *“I felt great because it made me more independent.”* Likewise, P34 (female, unconditional) stated: *“… it taught us how to be independent, and not to rely on boys.”* Adolescents described CTs as helpful, convenient, and providing access to resources that they needed. *“We need clothes; some of us use make-up, and we may need money to party with our friends; so, sometimes our parents can’t afford to give us money.”* (P4, female, clinic). Therefore, the cash provided them with resources that they would not otherwise have accessed.

Before the intervention, adolescents depended on their parents and family members, and some girls relied on their sexual partners for financial support. For most participants, this was stressful and caused conflicts, particularly in instances where funds were insufficient. When asked to describe her family relationships while receiving the CT, P38 (female, school), who lived with her sister responded: *“There are no [conflicts], because I don’t ask her [sister] for money. Sometimes ... you see, sometimes it’s bad when you start asking for money from people. If you don’t [ask] it’s okay.”*

Participants recognized the financial constraints faced by their parents, who were mostly single parents and held informal jobs or ran small businesses. Hence, they were mindful of putting extra financial pressure on their parents. Receipt of CTs lessened financial demands on participants’ parents. Moreover, to some extent, CTs provided resources that parents would have had to pay for; this meant that they could focus on other competing domestic needs, as the extract below indicates:

“It was one less stress for her. She was not worried about the matric dance anymore … She wasn’t going to be paying for it anymore. Because she was already worried about buying the books.” (P26, male, unconditional)

Feeling independent and responsible enhanced participants’ self-esteem and decision making, as P4 noted: *“It [CT] boosted many young peoples’ confidence by showing them that they can do things on their own without asking for help or working hard.”* CTs seemingly offered adolescents choices, including choices about their behavior. Some of the girls spoke about how receipt of CTs changed their behavior. P35, a young woman who received school-conditional payments, reported that, before the intervention, she counted on her boyfriend for financial support, since her mother was unemployed. As a way of showing appreciation to her boyfriend, she felt pressured to have sex with him. When she received CTs, she did not ask her boyfriend for money because she could support herself. She decided not to have sex with her boyfriend. She said:

“It helped me because I didn’t have to sleep with my boyfriend when I asked him for money … No, he never forced me to have sex with him, but I know that it makes him happy, you know … he said that if I want him to make me happy, then I must also make him happy.”

P35 continued to do things for herself, even after the intervention; she found part-time employment. Similarly, P1 stopped having multiple relationships and dating older men for financial support, and remained with one partner.

“The first one was the cash I received because it helped me stop doing many things that I used to do to get cash … Like dating older men, who usually gave me money to buy myself things.” (P1, female, school)

#### Cash management skills

Adolescents spoke about the gains that they had made from their involvement in the program. The majority mentioned that day-to-day administration of CTs equipped them with financial management skills. These skills included drawing up a budget, saving, and making independent financial decisions. *“It taught me things ... like, I can now budget for my money appropriately and know when to spend on the right things ...”* (P11, female, unconditional). Some participants reported saving their transfers over a short-term period for essential purchases or for an event that they wanted to attend. Participants felt in “control” and “responsible.” Some reported that had become open-minded, could help others, and had gained self-confidence.

### Developments after the intervention

Most adolescents, particularly those who had received a once-off single payment, reported that everything remained the same as it was prior to the program, stating that they knew that receipt of CTs was temporary. “*Nothing [changed]… I knew that I was going to get it once. It was like picking up money; you never know you are going to be lucky enough to pick it up again…”* (P21, male, clinic). Adolescents of both genders in the school and unconditional arm felt that a valuable resource had been taken away and that they are back to where they had started, relying on other people. This was stressful, as they had grown accustomed to having their own money and doing things for themselves, as the quotation below illustrates:

“Right now, everything that I come across that has to do with finances, I have to ask my parents, and sometimes this is difficult because they have their own things to pay for. So, I can say that my life has now changed, since I don’t have that money. Yeah, I can say that it went back to where I started. I’m used to having money I made myself.” (P40, male, unconditional)

Receipt of CTs appears to have somewhat compelled adolescent boys to earn an income. Two male participants reported that after the intervention, they saw a need to find employment, so as to meet their personal and families’ needs.

“I have to hustle to get money … When in the study, there was no need. But when the money stopped, there was a need because I still had those needs; so, I had to get more money to patch up that empty space.” (P28, male, unconditional)

The end of the CT program made a few of the boys think of easy ways of making money, such as engaging in criminal activities. However, only one criminal incident was reported.

“Money is an addiction for some people; so, the less they had, the more they wanted. Some had to try and steal other peoples’ goods to get the money for whatever they had to buy. I remember there was an incident by one person that stopped receiving the money; he mugged someone, took the person’s phone, and sold it to get money.”

One male participant (P45, male, school) reported that he was used to getting easy cash, money that he did not work for; so, after the program ended, he considered engaging in criminal activities to get quick cash without putting in much effort. It also appears that such thoughts and frustrations were experienced mostly by those who were neither in school nor employed after the program.

## Discussion

This qualitative study of CTs in a low-resourced, inner-city neighborhood explored adolescent recipients’ experiences and meanings of CTs. It also provides insight into adolescents’ spending patterns and outcomes after cessation of the CT program. This is important, as often long term outcomes after cessation of research studies are not known.

In our study context, CTs mostly generated positive experiences and meanings when transfers were offered directly to adolescents of both genders. Specific benefits included a sense of independence, financial freedom, and social growth; these attributes form a crucial component of adolescents’ development, as related to the transition from childhood to adulthood [29]. In low-middle income households, it is not always feasible for families to meet some adolescents’ needs, as these may be regarded as luxuries, in comparison to other competing household essentials. In our study, CTs enabled adolescents to purchase necessities such as toiletries, clothes, airtime, and make-up, and to pay for entertainment. CTs enhanced their financial stability and enabled them to participate in social and economic groups, thereby boosting their self-esteem. Increased self-confidence, autonomy, and decision-making among CT recipients influence women’s decisions to engage in protective sexual behavior [30, 31].

Most studies have shown that CTs offered to parents/guardians improved children’s outcomes [32]. However, we hypothesized that when beneficiaries are adolescents, the impact and outcomes may differ, as do adolescents’ needs from those of caregivers, resulting in adolescents using the money differently. In our study context, adolescents generally controlled the CTs and made decisions regarding expenditure; however, we observed some notable gender differences regarding these decisions. Boys were inclined to make independent decisions regarding expenditure, whereas girls discussed their expenditure plans with close family members; this is consistent with studies of adult women [33]. The extent of the independence with which adolescents made spending decisions might have influenced their spending and saving patterns. Although boys and girls generally spent the transfers in a similar manner, we observed some gender differences. Boys’ expenses reflected a concern with maintaining a social identity —to fit in with their peers— they were more likely to buy expensive clothing and spent the cash on socializing. These findings complement other research that found CTs enhanced men’s social status [34]. On the contrary, girls’ spending was more likely to reflect concern for domestic affairs and provision of support to other household members. This finding supports evidence showing that transfers directed to women tend to benefit the household and support family members.

While the adolescents in our study mostly reported positive expenditure; it is important to consider the implications of their expenditures in the local context. Research has shown high levels of alcohol consumption among young South African men [35]; thus, entertainment and socialization are likely to involve alcohol consumption by adolescent boys. Alcohol may not always elicit antisocial behavior; in fact, in some instances—as in our study—it enhanced men’s social status in the community; however, research shows that alcohol increases violence and the risk of HIV infection [36]. In our study, the issue of alcohol was raised by adolescents, as they spoke of friends or acquaintances who had spent CTs on alcohol. However, this did not emerge in the quantitative data, and only two young men reported personal use of CTs on alcohol. Additionally, regarding adolescent girls’ spending on domestic affairs, some argue that targeting women as beneficiaries of CT programs may perpetuate traditional gender roles, adding to the existing domestic responsibilities that women already carry [37].

The sustainability of the CT program’s effects after the program’s cessation remains questionable. In our study, the benefits to adolescents’ appeared to be short–lived, as they reverted to old behaviors and relying on their parents; this was probably because of the short intervention period. In a few instances, the intervention introduced pressure to earn money, particularly among boys. Pressure to make money may be either a positive or an adverse outcome; positive in the sense that, after the program had ended, some adolescents had also completed schooling, so they had found employment to sustain themselves. However, for others, the program created a need to make easy money, and could lead to committing crime as reported by two participants.

The experience of the program and its processes, conditionalities, cash delivery strategies, and the grant size were acceptable to the majority of boys and girls. Our findings show that conditionalities based on routine activities such as school attendance was not felt, since they do not require additional effort, unlike if unusual or dreaded activities had been set as conditions. However, if conditions include activities that adolescents consider necessary (i.e., knowing one’s health status), then CTs are more likely to encourage uptake of the dreaded service. Contradictory to other studies that reported jealousy and tension between recipients of CTs and participants in the control group [38], no adverse outcomes for social relationships were reported in our study. This could be because we conducted the project in a limited number of schools, and not many people in the participants’ neighborhoods knew about the study. Moreover, all participants in the trial received transfers.

### Study limitations

This study was not without limitations. We acknowledge that interviewing a different set of individuals at 12 months follow-up limited the depth of understanding of individual experiences. Participants provided self-reports on how the transfers were used; thus, there is potential for bias in their reports of their spending patterns, with the possibility that responses perceived as desirable were provided. For example, although the majority of participants in the school condition reported positive outcomes we cannot rule out the potential social desirability bias particularly because the findings from the main trial indicate that participants in the school conditioned study arm were more likely to miss school.

## Conclusion

Our findings demonstrate that from the perspective of young men and women, adolescent-directed CT programs are acceptable and benefit individuals as well as households in low-resourced urban environments. They instill a sense of agency and responsibility in adolescents, facilitating transition to adulthood. The gender differences in adolescents’ spending decisions and expenditure regarding CTs are worth noting. Spending on domestic domains in girls and public domain in boys show that CTs could potentially reaffirm traditional gender norms in this population. Program implementers need to take this into consideration, and think strategically about targeting adolescent girls with the provision of CTs. However, we also recommend that boys be considered for CT programs. Despite no data being available on the effects of CTs on HIV prevalence or incidences among males, access to transfers contributes towards their psychosocial development, although this needs to be balanced against the risks of expenditure on alcohol and alternative money-making strategies that could lead to consideration of criminal activities when the CT program ends. These, however, require further exploration. These findings provide additional insights to studies that highlight the importance of adding more supplemental funds to the already existing social grants for vulnerable children in South Africa.

## Abbreviations

ART: Antiretroviral therapy
CCT: Conditional Cash Transfer
CT: Cash transfer
PMTCT: Prevention of mother-to-child transmission
RCT: Randomized Controlled Trial
UNDP: United Nations Development Program
